# The Role of CD1d and MR1 Restricted T Cells in the Liver

**DOI:** 10.3389/fimmu.2018.02424

**Published:** 2018-10-30

**Authors:** Wenyong Huang, Wenjing He, Xiaomin Shi, Xiaoshun He, Lang Dou, Yifang Gao

**Affiliations:** Organ Transplantation Unit, First Affiliated Hospital of Sun Yat-sen University, Guangzhou, China

**Keywords:** iNKT cells, MAIT cells, liver diseases, innate T cells, CD1d restriction

## Abstract

The liver is one of the most important immunological organs that remains tolerogenic in homeostasis yet promotes rapid responses to pathogens in the presence of a systemic infection. The composition of leucocytes in the liver is highly distinct from that of the blood and other lymphoid organs, particularly with respect to enrichment of innate T cells, i.e., invariant NKT cells (iNKT cells) and Mucosal-Associated Invariant T cells (MAIT cells). In recent years, studies have revealed insights into their biology and potential roles in maintaining the immune-environment in the liver. As the primary liver-resident immune cells, they are emerging as significant players in the human immune system and are associated with an increasing number of clinical diseases. As such, innate T cells are promising targets for modifying host defense and inflammation of various liver diseases, including viral, autoimmune, and those of tumor origin. In this review, we emphasize and discuss some of the recent discoveries and advances in the biology of innate T cells, their recruitment and diversity in the liver, and their role in various liver diseases, postulating on their potential application in immunotherapy.

## Introduction

The liver is a primary internal organ that plays a unique role in pathogen defense. Approximately 1/3 of total blood passes through the liver every minute (1, 2). Once the blood enters, it circulates at a reduced flow rate through the sinusoids, which comprise a complex vascular network of capillary-like vessels. The reduced flow rate maximizes the opportunity for pathogens to recognize the hepatic immune environment.

The ability to restrict and eliminate invading pathogens is one of the main features of the immune system. Much hepatology literature has focused on the adaptive, antigen-specific classical T-cell populations and their role in the protection and pathogenesis of liver disease. However, human liver is selectively enriched in innate T cells, including natural killer T (NKT) cells (3) and Mucosal-Associated Invariant T (MAIT) cells (4, 5). These innate T cells are unconventional T cells with diverse functions that play an essential role in liver immune surveillance. Similar to other innate cells, recognition of antigens from microbial, endogenous glycolipids or metabolites activates innate T cells, allowing them to produce cytokines and cytolytic proteins (4, 5). One of the most important features of innate T cells is bridging the innate and adaptive immune response, and various types of cytokines produced after innate T cell activation can modulate CD4^+^ and CD8^+^ T cell immune response (6). During liver injury, innate T cells infiltrating into the inflammatory site after neutrophils and monocytes are proposed to be sensors that control the local immune response (7). Although innate T cells have previously been defined primarily by phenotypic markers, recent emerging evidence has revealed considerable functional complexity in this population. This review summarizes the current literature regarding iNKT, type II NKT and MAIT cells, which have important roles in a variety of liver diseases, particularly focusing on their role in human liver diseases.

## The biology of innate T cells

**Figure 1:**
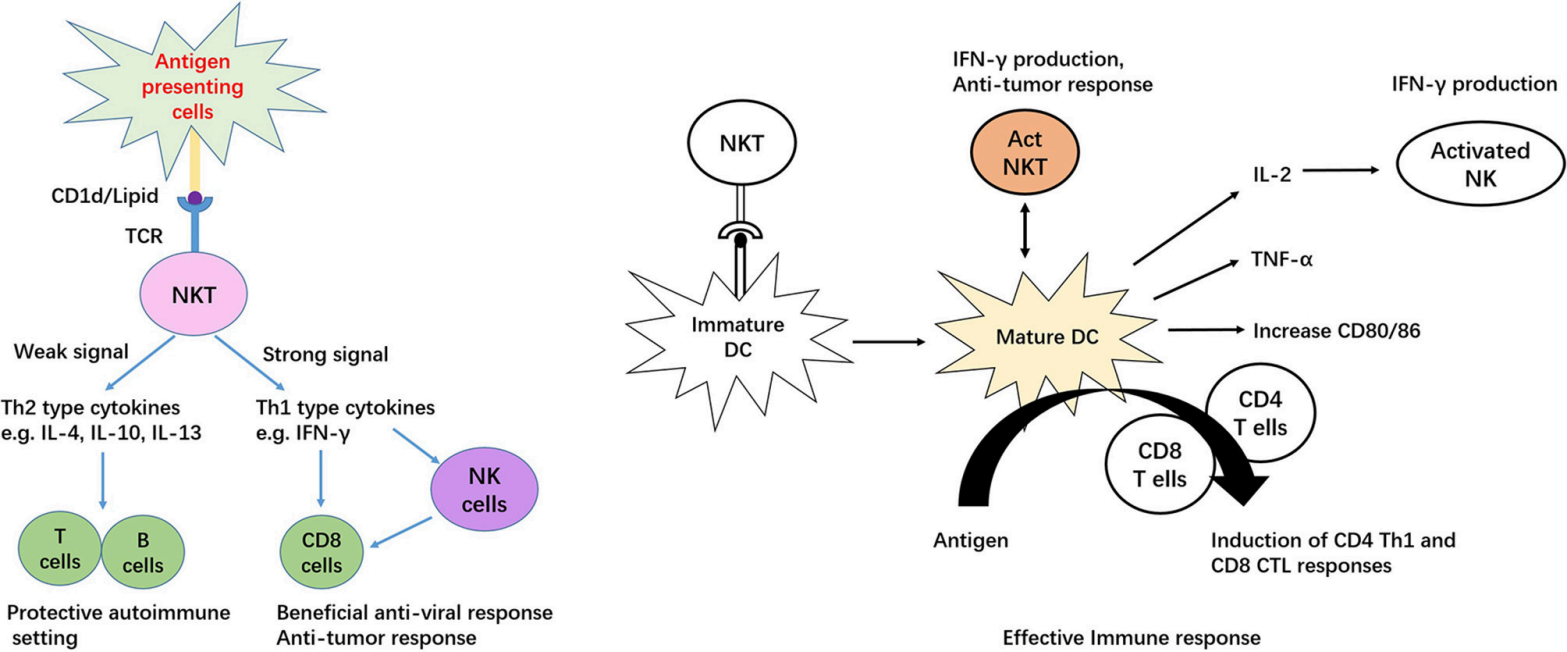
The dual role of iNKT cells as immune regulators.

iNKT cells, also known as type I NKT cells or classic NKT cells, are a subset of natural killer T (NKT) cells, while another subset of NKT cells is type II NKT cells. iNKT cells are characterized by signatures of both T and NK cells, including broad range expression of molecular markers that are typically associated with NK cells, for example, NK1.1 in mouse and CD161/CD56 in human (8, 9). Notably, these markers alone may be insufficient to distinguish iNKT cells, because in mouse, NK1.1 is not expressed in certain strains, including AKR, BALB/c, CBA/J, C3H, DBA/1, DBA/2, NOD, SJL, and 129 (10). There are reasonable amounts of MAIT cells in human peripheral blood and liver that also express CD56 (11); therefore, co-staining with CD1d tetramer should precisely identify iNKT populations. The so-called invariant is based on limited TCR arrangement, Vα14Jα18/Vβ2, Vβ7, and Vβ8 in mouse and Vα24Jα18/Vβ11 in human (12, 13). Mouse and human iNKT cells can recognize lipid and glycolipid antigens of self or microbial origin presented on MHC class-I-like CD1d molecules (14, 15). Activation by the agonist α-galactosylceramide (α-Galcer) allows mice and human iNKT cells to readily proliferate, undergoing significant remodeling of their surface expression patterns with regard to several markers, such as NK1.1 and the semi-invariant TCR, resulting in production of abundant Th1, Th2 and Th17 type cytokines, including IFN-gamma, IL-4, IL-13, and IL-17 (16–18). Apart from the TCR dependent pathway, human iNKT cells can recognize and eliminate target cells expressing NKG2D ligands in a TCR-independent manner (19). Cytokines released by stimulated iNKT cells are able to transactivate other innate and innate-like immune cell subsets, thereby amplifying their initial responses (20–24). In addition, iNKT cells provide both antigen-specific cognate and non-cognate aid to B cells (25) and in turn, are activated by B cells (26, 27). Interestingly, unlike the non-cognate iNKT cell–B cell interactions Figure 1, antigen-specific cognate iNKT cells induce a more innate-biased B cell response characterized by a discontinuous germinal center B cell expansion and rapid initial proliferation of IL-10-producing B cells that fails to induce humoral memory (28).

There are three functional subsets of iNKT cells in mouse and human, which produce a distinct combination of cytokines and lineage-specific transcription factors, namely, NKT1, NKT2, and NKT17. Murine studies have demonstrated that T-box 21 (T-bet), GATA binding protein-3 (GATA3), and retinoic acid receptor-related orphan nuclear receptor gamma (RORγt) are expressed on iNKT1, iNKT2, and iNKT17 cells, respectively, and these transcription factors are correlated with the function of iNKT cells (29). Mirroring T helper cell subtypes, iNKT1, iNKT2, and iNKT17 cells produce IFN-γ, IL-4, and IL-17 (29). These cytokines allow iNKT cells to interact with other immune cells. For example, IL-4 produced by NKT2 cells at steady state through phosphorylation of signal transducer and activator of transcription 6 (STAT6) regulates CD8 T cells developing to a memory-like phenotype in the thymus (30). Additionally, IL-4 promotes antibody production by B cells and induces dendritic cells to secrete T helper (Th) 2-type chemokines, such as chemokine (C-C motif) ligand (CCL) 17 and CCL22 (30). Moreover, in lymphoid organs, each iNKT subset displays different anatomic localization, which determines their responsiveness to intravenous or oral antigenic challenges (31). Recently, RNAseq analysis has suggested that each iNKT subset has a unique genetic signature, and these footprints are more similar to γδ T cells and innate lymphoid cells (ILCs) than to conventional T cells (32).

Compared to iNKT cells, type II NKT cells express relatively diverse TCRs that can recognize antigens derived from microbial, endogenous glycolipids, phospholipids and endogenous hydrophobic peptides presented by CD1d molecules (33). Type II NKT cells are unable to recognize α-linked glycolipids, for example, α-Galcer, but are responded to β-linked glycolipids (34). Igor Maricic et al. found that mice type II NKT cells are activated by self-phospholipids, for example, lysophosphatidylcholine (LPC), lysosphingomyelin (LSM) and lyso-platelet-activating factor (LPAF) (34). According to their function, type II NKT cells can be divided into pro-inflammatory and anti-inflammatory subsets (34). Stimulating type II NKT cells with self-glycolipid sulfatide inhibits inflammatory responses induced by CD4^+^ T (35) and iNKT cells (36) in mice. In a mouse bone marrow transplantation model, donor type II NKT cells inhibit graft-vs.-host disease via releasing IL-4 (37). In a rat model, activating type II NKT cells with SCP2 peptide promotes the inflammatory response through production of inflammatory cytokines IL-5 and IL-6 (38). In an iNKT cell-deficient (Jα18^−/−^) mouse model, Sagami et al. found adoptive transfer of type II NKT cells exacerbated DSS-induced colitis (39).

MAIT cells are a subset of αβ T cells that possess both innate and effector-like qualities (40, 41). They are preferentially located in the gut lamina propria and express an invariant α chain (42). Similar to other αβ T cells, MAIT cells undergoing conventional TCR arrangement express canonical Vα7.2-Jα33 TCRs paired with variable β-chains in human (43, 44) and Vα19-Jα33 TCR in mice (42). In general, MAIT cells are equipped with effector properties before exiting from the thymus (45, 46). These cells were initially discovered as invariant α chain-expressing cells in the double-negative T cell fraction of human peripheral blood by Steven Porcelli et al. in 1993 (47). Later studies found that MAIT cells express high levels of CD161 and are also present in CD4-positive, as well as CD8-positive, lymphocytes (48).

Distribution of MAIT cells differs between humans and mice. The frequency of MAIT cells in C57BL/6 mice accounts for ~0.1% of the peripheral T cell population (49), whereas 1–10% of them are identified in human peripheral blood. Moreover, the occurrence of MAIT cells varies widely among tissues in healthy adults, ranging from 2% (ileum) to 60% (jejunum), and they can make up ~20–50% of intrahepatic T cells.

Interestingly, MAIT cells are absent in germ-free mice, indicating that their expansion in the periphery depends on the presence of microbial ligands (40, 42). MAIT cells are MR1, an MHC class I-like protein, restricted-T cells. Recognizing bacterial-produced vitamin B metabolites presented by MR1 allows MAIT cells to secrete a vast amount of pro-inflammatory cytokines, including IFN-γ, TNF-α, IL-2, and IL-17 (11, 41, 50), which lyse bacterially infected cells (51, 52). Additionally, comparative genomic analysis has demonstrated that MR1 is only expressed in marsupial and placental mammals and is exceptionally highly conserved, particularly at the α1 and α2 domains of ligand-binding grooves (53). Both MR1 and MAIT TCR genes are extremely highly conserved, implying that evolutionary pressure is involved in maintaining conservation of these genes (42).

MAIT cells can defend against microbial activity and infections caused by bacteria or yeast through activating the vitamin B2/riboflavin pathway in an innate manner (42, 45, 54). A study by Michael S. Bennett et al. provided evidence that supernatants from stimulated human MAIT cells promote B cell plasmablast differentiation and IgA, IgG, and IgM production (55). Additionally, human MAIT cells respond to mycobacterium tuberculosis infection and provide an early source of IFN-γ required for activation of the Th1 response (56). Together, these results indicate the potentially important role of MAIT cells in the defense against microbial invasion.

## CD1D restricted T cells in liver health and disease

iNKT cells contribute to a significant subset of lymphocytes in the liver. In mice, iNKT cells are most abundant in the liver (10–30%), with lower frequencies found in the thymus, blood, bone marrow and lymph nodes (0.1–0.2%). In humans, high iNKT cell numbers are detected in the liver (1%) (57, 58), compared to 0.01–0.5% in their peripheral counterparts (59, 60). The distribution of type II NKT cells is difficult to investigate due to their lack of specific surface markers (61). The literature suggests that type II NKT cells may outnumber iNKT cells in humans (61).

**Figure 2:**
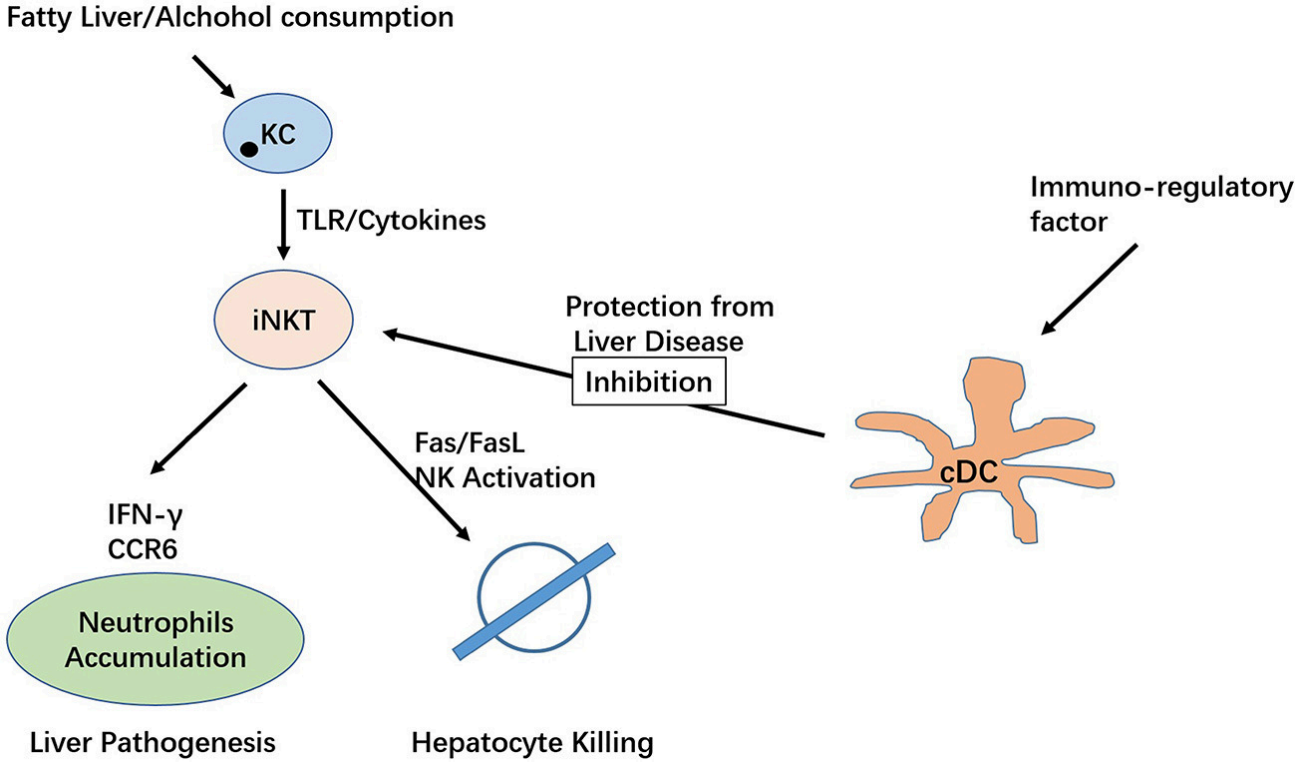
The protective and pathogenic roles of iNKT cells in the liver.

iNKT cells mediate various functions in the liver, including hepatic injury, fibrogenesis, and carcinogenesis. As one of the important immune subsets in the liver, iNKT cells demonstrate a pathogenic role in IRI, primary biliary cirrhosis, non-alcoholic fatty liver, and hepatitis. Interestingly, a protective role was identified for these cells in an acute liver injury model (Figure 2). To date, literature appears to suggest that iNKT cells exert a protective role during the acute phase of liver injury and a pathogenic role in chronic conditions (62). Some attention has also been focused on the implication of NKT cells in liver transplantation, including their role in ischaemia/reperfusion injury and transplantation rejection (63, 64). The study of type II NKT cells in liver disease progression is limited, focusing on hepatitis viral infection, where type II NKT cells appear to play a controversial role in controlling liver injury. A new subset of type II NKT cells, II NKT-Tfh cells, have been found to regulate metabolic lipid disorders (65).

### Hepatocellular carcinoma

HCC is often linked to chronic inflammatory liver diseases, such as NASH and viral hepatitis (66). A murine model suggested that TLR4 and canonical nuclear factor-κB signaling in the liver facilitate NASH-to-HCC conversion. Fundamentally, iNKT cells have a dual role in cancer that either promotes an anti-tumor response or elevates tumor growth via activation of effector T cells promoting Th1 responses or recruitment of regulatory T cells to induce Th2 responses (67, 68). Although hepatic iNKT cells are rich in number, relatively few studies have attempted to clarify their role in HCC, and results from these studies appear contradictory.

HCC patients exhibit increased iNKT cell numbers in the tumor site compared to the peripheral blood. More importantly, hepatic iNKT cells are found to secrete Th2 cytokines, thus inhibiting tumor-specific CD8^+^ T-cell responses (66, 69). In contrast, murine studies identified that CD4^+^ iNKT cells could mediate anti-tumor responses through inhibition of the inflammatory response triggered by activation of the oncogenic β-catenin pathway (70). Additionally, iNKT cells are able to suppress tumor growth after adoptive transfer of HCC tumor lines in mice (71, 72).

Very recently, a study by Ma et al. found that commensal bacteria are important regulators of anti-tumor immunity that alter hepatic natural killer T cells. This regulation strengthens IFN-gamma production by hepatic natural killer T cells and promotes anti-tumor effects (73).

Current studies have suggested that type II NKT cells may play an immune regulatory role in cancer settings. In CD1d knockout and Jα18 knockout mice, Terabe et al. found that activation of CD1d-restricted type II NKT cells is sufficient for downregulation of tumor immunosurveillance in mouse fibrosarcoma, mammary carcinoma, colon carcinoma, and lung metastases of the CT26 colon carcinoma models (74). Using the same knockout method, Renukaradhya et al. demonstrated that type II NKT cells supress anti-tumor immunity against B-cell lymphoma (75). In addition, there are higher frequencies of IL-13 releasing type II NKT cells in myeloma patients than in healthy donors (76).

### Hepatitis viral infection (HCV and HBV)

Overall, 57% of liver cirrhosis cases and 78% of liver cancers are caused by chronic HBV and HCV infections, accounting for almost a million deaths every year. A few studies have attempted to identify the role of iNKT cells in controlling HCV infections, in particular, during the initial phase of infection. In human hepatic CD3^+^CD56^+^ cells, including iNKT cells, HCV replication is inhibited in hepatocytes by IFN-γ secretion, and this activity is positively correlated with disease progression (77). Furthermore, it modulates the effectiveness of IFN-alpha in late HCV infection. However, studies have shown that iNKT cells are considerably depleted in chronic HCV infection (78–80). This finding may suggest that iNKT cells contribute to the early phase of HCV infection but not as much to disease progression.

Similarly, high numbers of activated type I NKT cells have been identified in the early stages of HBV infection in humans (78–80). In agreement with those results, CD1d expression is elevated in HBV+ve liver tissue compared to HBV–ve counterparts (81). Similar to their action in HCV infections, the inhibitory effect of iNKT cells on HBV occurs through secretion of IFN-γ as well, activating the adaptive immune response and inhibiting viral replication (82). Recently, Xu and colleagues have shown that exhaustion marker Tim-3 is upregulated on hepatic iNKT cells from HBV-transgenic mice (83). Blockade of Tim-3 by anti-Tim-3 antibody strongly enhances expression of IL-4, IFN-γ, TNFα, and CD107a in iNKT cells and augments α-Galcer-induced inhibition of HBV replication (83). Interestingly, researchers have found that on the one hand, iNKT cells control the replication of hepatic viruses, while on the other hand, they contribute to virally induced liver injury through production of pro-inflammatory cytokines that induce hepatocyte apoptosis and inhibit proliferation (84–86).

The function of type II NKT cells in hepatitis viral infection is debatable. In a ConA-induced mouse hepatitis model, activation of type II NKT cells with sulfatide or LPC evoke anergy in iNKT cells that suppresses inflammation-triggered liver damage (34). In contrast, hepatic type II NKT cells promote development of liver injury in a transgenic mouse model of acute hepatitis B virus infection (87). Stimulation of type II NKT cells triggers conventional T-cell activation and pro-inflammatory cytokine production, resulting in augmentation of hepatic injury in murine autoimmune hepatitis models (88).

### Non-alcoholic fatty liver disease

In the modern era, NAFLD is considered the most frequent chronic liver disease in developed countries, affecting ~10–20% of the population. NAFLD is characterized by abnormal accumulation of fat in the liver, leading to infiltration of inflammatory cells accompanied by fibrosis or necrosis progressing to liver cirrhosis or hepatocellular carcinoma (HCC) (89, 90). Current studies on systemic analysis of iNKT cell subsets in non-alcoholic fatty liver disease are very limited, with a few studies confirming their importance. In human hepatic CD1d cells, the number of CD3^+^CD56^+^ cells are elevated in NASH patients (91). Reduced iNKT cell counts were found in mice fed high-fat diets and in obese mice (92, 93). In addition, mice with NAFLD lacking iNKT cells showed increased pro-inflammatory mediator factor and increased levels of TLR4 and PDGF2 mRNA (94). Activation of Kupffer cells (KCs) could cause apoptosis in these cells and further contribute to steatosis and insulin resistance (92, 95). Depletion of KCs could reduce hepatic IL-12 expression and rescue iNKT cells from apoptosis, preventing further pathological changes in the disease. Tim-3/galectin-9 is known to regulate the homeostasis of liver iNKT cells in the murine system (96).

Indeed, depletion of KCs via treatment with gadolinium chloride reduces hepatic IL-12 expression and does not lead to iNKT apoptosis, thereby preventing diet-induced hepatic steatosis and insulin resistance. Consistently, activation of the Hedgehog pathway and HSCs have been revealed to be associated with iNKT cells in mice fed an MCD diet or a combination of a CD-HFD (97–99). Using a diet-induced mouse obesity model, Satoh and colleague show that type II NKT cells trigger inflammation in the liver and exacerbate obesity (100).

### Alcoholic liver disease

ALD is caused by chronic alcohol abuse resulting in alcoholic fibrosis or cirrhosis. The disease currently one of the most frequent causes of death. Activation of KCs via LPS/TLR signaling-dependent mechanisms following alcohol consumption result in increased secretion of a variety of pro-inflammatory cytokines and chemokines, in addition to eicosanoids and reactive oxygen species (101, 102). Mechanisms underlying ALD include a complex network of hepatocytes, KCs, DCs and innate T cells (103). Studies found activation of KCs via the LPS/TLR pathway following alcohol intake, which increases secretion of a variety of pro-inflammatory substances (101, 102, 104). In a murine model, increasing TNFα and IL-1β production were observed in alcohol-fed mice that neutralize IL-1β in KCs to allowed iNKT cell accumulation and steatosis. The study also demonstrated that NLRP3 inflammasome and IL-1β secretion are essential factors for hepatic iNKT cells to accumulate and activate in ALD (105). Consistently, gut microbes can also trigger KC NLRP3 activation, resulting in iNKT cell activation (105). A study by Mariric et al. examined the role of both type I and type II NKT cells in alcoholic liver disease, demonstrating that only iNKT cells became activated following heavy alcohol consumption, resulting in inflammation and liver tissue damage. This study suggests that type I and II NKT cells are functionally distinct in liver inflammation and tissue injury (106). A more recent study identified the interplay between IL-10-producing iNKT cells silencing the productive roles of NK cells in alcoholic liver disease (107).

Despite these findings, the role of iNKT cells in human ALD has not been well-examined. In agreement with the murine data, pro-inflammatory cytokine levels were increased in alcoholic hepatitis human subjects, suggesting a correlation with disease severity (57). In addition, in patients with alcoholic hepatitis, NKG2D expression in NK and iNKT cells has been found to correlate with disease severity, suggesting these cells are involved in promoting liver damage (108).

## MAIT cells in liver health and disease

**Figure 3:**
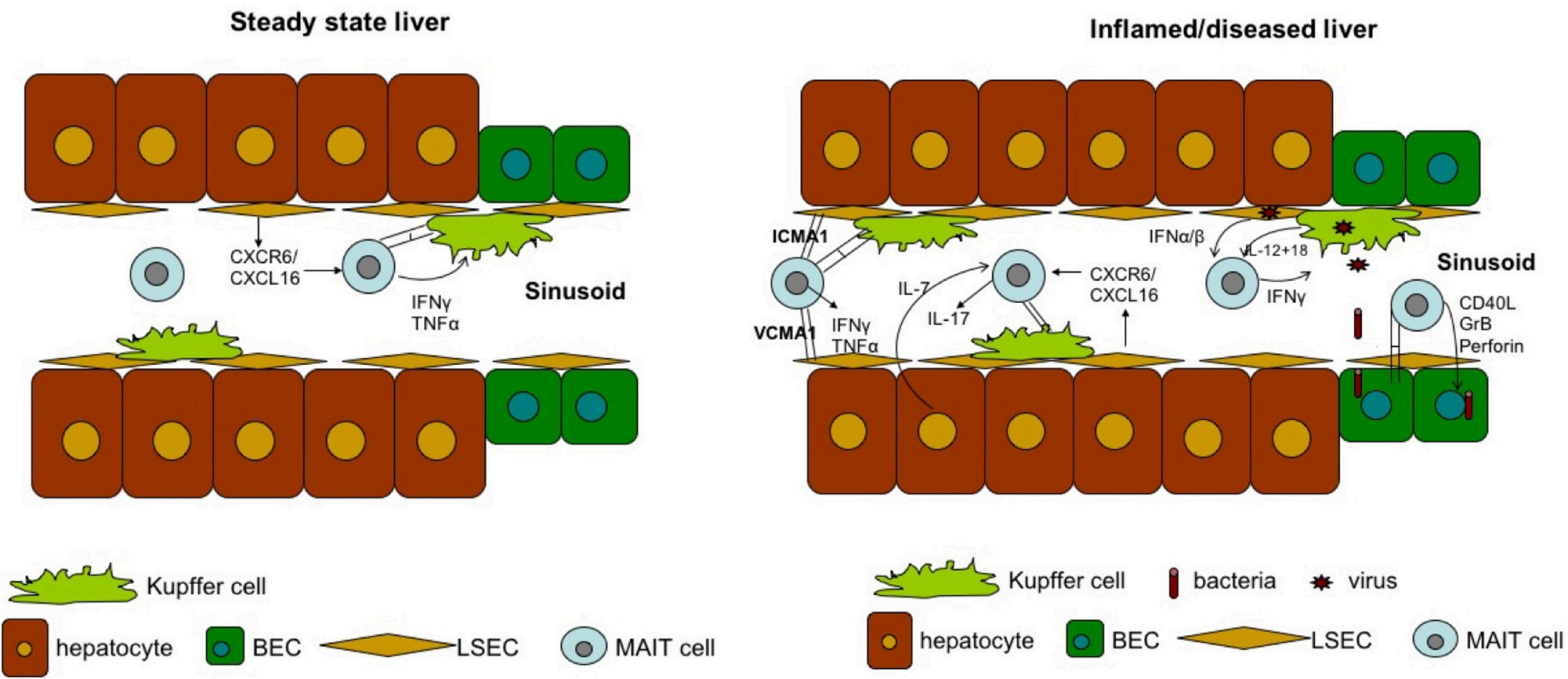
The role of hepatic MAIT cells during steady state (left) and disease state (right).

MAIT cells are significantly enriched in the liver, where they comprise up to 50% of liver-resident lymphocytes. These cells are located primarily in the biliary tract, and in the context of liver infection, MAIT cells can be activated by MR1-presenting bacterial ligands or indirectly via IL-12 and IL-18 produced by antigen-presenting cells in response to Toll-like receptor 8 signaling triggered by viral RNA (109, 110). The importance of MAIT cells in liver immunosurveillance is highlighted by three findings. First, liver MAIT cells are highly activated and express the activation marker CD69, as well as HLA-DR and CD38 (11, 110). This activation status suggests that liver MAIT cells are in a highly activated state, poised to respond to incoming antigens from the gut. Second, intra-hepatic MAIT cells, along with CD56bright NK cells, are the main source of IFN-γ post-TLR8 stimulation by liver-derived mononuclear cells through IL-12 and IL-18 activation (80). Finally, MAIT cells are the predominant IL-17 producers among intrahepatic T cells (~65% of IL-17^+^ T cells) in response to phorbol 12-myristate 13-acetate/ionomycin stimulation (11). As IL-17 targets multiple cell types in the liver, including Kupffer cells and BECs, to produce pro-inflammatory cytokines and chemokines (111), MAIT cells may be important regulators of hepatic inflammation and fibrosis Figure 3.

### Hepatocellular carcinoma

Recent studies have found that MAIT cells are recruited from peripheral blood to solid tumors in several cancers (112–114). Infiltration and accumulation of MAIT cells into tumor sites suggest that MAIT cells play an essential role in tumor development. MAIT cells are highly enriched in human liver (109) but are resisted to skew to an IL-17-producing phenotype, as they fail to release to IL-17 upon TCR stimulation (11). On the other hand, the function of tumor-infiltrating MAIT cells is proposed to be impaired in response to a panel of TCR ligands and cytokines (115). In a colorectal liver metastasis setting, IFN-γ produced by hepatic tumor-infiltrating MAIT cells is significantly suppressed (115). Taken together, the role of MAIT cells in hepatocellular carcinoma is still obscure, and further studies investigating the phenotype of tumor-infiltrating MAIT cells and their interactions with liver-resident cells will help to understand the role of MAIT cells in HCC.

### Hepatitis B virus (HBV)

To date, the role of hepatic MAIT cells in HBV is still poorly understood. Two studies have compared peripheral MAIT cells in healthy controls and chronic HBV patients, showing opposing results. The first study found that MAIT cells were not deleted nor functionally impaired in HBV patients (116). In contrast, there was a higher frequency of MAIT cells expressing CD38 and releasing granzyme B in HBV patients, suggesting that MAIT cells were more activated in the HBV setting (116). The second study demonstrates that in HBV patients, MAIT cells are in an exhausted phenotype, where the frequency of cells in circulation is reduced, the expression of the early activation marker CD69 is inhibited, and the production of IFN-γ and granzyme B are significantly suppressed (43). Why there are discrepancies between these two studies is not clear. The opposite observations on granzyme B production may be explained by different activation methods, as Boeijen et al. activated MAIT cells with IL-12/IL-18/CD28^+^ *Escherichia coli* (116), while Yong et al. stimulated MAIT cells with PMA/ionomycin (43). It should be taken into consideration that the size of both studies is relatively small. Therefore, the patients could have been in various clinical phases and undergoing different treatments. Indeed, MAIT cells are abundant in the peripheral blood but account for only a small percent of T cells (1–10%) (117). MAIT cells are further enriched in the liver (20% to 50% of T cells), which is also the primary site of infection (117). Therefore, further research with larger cohorts that focus on intrahepatic MAIT cells is required to solve the mystery of MAIT cells in HBV.

### Hepatitis C virus (HCV)

Several studies have shown that CD8^+^, rather than CD4^+^, MAIT cells in the peripheral blood were significantly reduced in the setting of chronic HCV (118, 119). These results may be due to CD8^+^ MAIT cells belonging to a newly defined pro-apoptotic phenotype expressing high levels of caspase 3 and 7 (120). Further phenotypic and functional studies reveal that the remaining CD8^+^ MAIT cells represent a chronic activation phenotype with signs of immune exhaustion, which is characterized by elevated levels of CD38, HLA-DR, CD69, PD-1, TIM-3, CTLA-4, and Granzyme B (118, 119). Notably, the function of these MAIT cells is also impaired, as reflected by the production of IFN-γ and TNFα being actively suppressed upon stimulation with TCR-dependent *E. coli* but not TCR-independent IL-12^+^IL-18 (118, 121). This result suggests that the loss and functional impairment of MAIT cells is a non-reversible process in chronic HCV patients, as antiviral treatment cannot reinvigorate these MAIT cells (118, 121, 122). Arguably, Ben Youssef et al found that adult MAIT cells in peripheral blood expand from cord blood Vα7.2^+^ CD161^high^ T cells, and this process lasts ~5 years before filling up the adult MAIT pool (123). Therefore, the dysfunction and loss of MAIT cells after antiviral therapy may be due to the slow kinetics of differentiation and proliferation in MAIT cells.

There is an inverse correlation between the frequency of hepatic MAIT cells with liver inflammation and liver fibrosis in the setting of chronic HCV, demonstrating that MAIT cells are crucial mediators against HCV infection in the liver (121). Similarly, the percentage of hepatic MAIT cells is also reduced in chronic HCV patients (121). Importantly, the expression of HLA-DR and CD69 on MAIT cells is higher in the liver, suggesting that intrahepatic MAIT cells are more activated than are peripheral MAIT cells (121). This difference may because there is a higher frequency of activated monocytes in the liver, as they are an important source of IL-18 (121). MAIT cells are deleted in both blood and liver in the setting of HCV, and it is hypothesized that blood MAIT cells migrate to the organ, where they are further stimulated by inflammatory cytokines, resulting in activation-induced death, a mechanism that has been observed and well-characterized in HIV-induced MAIT cell depletion (121, 124).

### Non-alcoholic fatty liver disease

The major cause of NASH/ NAFLD is chronic liver inflammation induced by tissue damage or pathogen infection (125). Hegde et al. finds that the number of hepatic MAIT cells is decreased in patients with non-alcoholic fatty liver disease-related cirrhosis (126). Compared with controls, cirrhotic liver MAIT cells exhibit an activated phenotype characterized by increasing IL-17 production with no differences in the percentage of MAIT cells producing granzyme B, IFN-γ, or TNF (126). Another study demonstrated that MAIT cells in NASH patients also display an activated phenotype defined by enhanced cytotoxicity but reduced cytokine production (127). These experiments suggest that MAIT cells are activated and contribute to pathogenesis in NAFLD/NASH.

### Alcoholic liver disease

One of the most frequent complications of ALD is bacterial infection. One study has shown that over 50% of severe alcoholic hepatitis patients suffer from bacterial infection (128). As potent antibacterial lymphocytes in the liver, the number, cytokine production (IL-17) and cytotoxic response (Granzyme B, CD107a) of MAIT cell are impaired in peripheral blood of severe alcoholic hepatitis and alcoholic cirrhosis patients (129). Dysfunction of MAIT cells in ALD patients occurs from exposure to bacterial antigens and metabolites, but not ethanol (129). Importantly, in the liver, microarray data show that expression of transcription factors RORC/RORγt, ZBTB16/PLZF, and Eomes that mediate the function of MAIT cells is lower in ALD patients than in controls (129). Together, these results suggest that MAIT cells in ALD display a defective phenotype, which may explain why there is a high rate of bacterial infection complications in ALD patients.

## Conclusions

Herein, we have discussed several key aspects of innate T cells and their potential role in liver diseases. Their enrichment in the liver suggests their unique role in liver disease progression and protection. The distinctive features and functions of innate T cells impart both pathogenic and protective abilities to the host. Thus, modulation of these cells represents a very attractive therapeutic strategy in liver diseases.

Our current knowledge of these cell subsets in the liver and their potential role in liver disease mainly comes from studies in animal models. Data from human and clinical studies are insufficient and are primarily complicated by the opposing effect these cell types have, both pathogenic and protective. In addition, most liver diseases are chronic disorders, and this further complicates analysis of these cells. The dynamic effect of these cells at different time points during the progression of disease could be significantly different regarding both number and function. Another critical factor is that a vast number of immune-regulatory cells resides in the liver, where they all modulate the activity level of iNKT and MAIT cells. In turn, these two innate T-cell subtypes also act as key modulators of other immune cell activity, including KCs, classical innate cells (macrophages and DCs) and conventional T cells. These factors form an involved local liver environment; thus, a molecular understanding of these cross-regulatory effects is key to understanding the liver immune system. Understanding liver immunity and function is also key to maintaining a proper balance between immune tolerance and immunity in the liver. To further understand the mechanism of these cells, it will be essential to develop more specific and reliable reagents to characterize and analyse these cells.

## Author contributions

WHu, WHe, XS, LD, XH and YG contributed to the writing of the manuscript.

### Conflict of interest statement

The authors declare that the research was conducted in the absence of any commercial or financial relationships that could be construed as a potential conflict of interest.
